# Proposed evolutionary changes in the role of myelin

**DOI:** 10.3389/fnins.2013.00202

**Published:** 2013-11-08

**Authors:** Klaus M. Stiefel, Benjamin Torben-Nielsen, Jay S. Coggan

**Affiliations:** ^1^The MARCS Institute, Sydney, University of Western SydneyNSW, Australia; ^2^The Blue Brain Project, Ecole Polytechnique Fédérale de LausanneLausanne, Switzerland; ^3^Department of Neurobiology, Hebrew University of Jerusalem, Edmond J. Safra CampusJerusalem, Israel; ^4^NeuroLinx Research InstituteLa Jolla, CA, USA

**Keywords:** myelin, evolution, Mesozoic marine revolution, brain energy consumption, axonal conduction speed

## Abstract

Myelin is the multi-layered lipid sheet periodically wrapped around neuronal axons. It is most frequently found in vertebrates. Myelin allows for saltatory action potential (AP) conduction along axons. During this form of conduction, the AP travels passively along the myelin-covered part of the axon, and is recharged at the intermittent nodes of Ranvier. Thus, myelin can reduce the energy load needed and/or increase the speed of AP conduction. Myelin first evolved during the Ordovician period. We hypothesize that myelin's first role was mainly energy conservation. During the later “Mesozoic marine revolution,” marine ecosystems changed toward an increase in marine predation pressure. We hypothesize that the main purpose of myelin changed from energy conservation to conduction speed increase during this Mesozoic marine revolution. To test this hypothesis, we optimized models of myelinated axons for a combination of AP conduction velocity and energy efficiency. We demonstrate that there is a trade-off between these objectives. We then compared the simulation results to empirical data and conclude that while the data are consistent with the theory, additional measurements are necessary for a complete evaluation of the proposed hypothesis.

## Introduction

**Figure 1:**
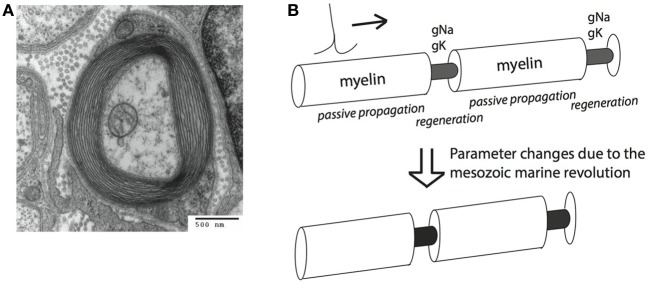
**Myelin sheets engulf axons and can increase conduction speed and energy efficiency of action potential (AP) conduction. (A)** An electro-micrograph of a myelin sheath. Image by the Electron Microscopy Facility at Trinity College via Wikipedia. **(B)** Schematic representation of salutatory AP conduction. Different parameters are optimal for conduction speed (bottom) and energy efficiency (top) of action potential conduction. Our hypothesis is that the Mesozoic marine revolution led to a transition between these different parameter sets.

Myelin is a neural cellular specialization unique to vertebrates (with a few interesting exceptions, Davis et al., 1999; Hartline and Colman, 2007). It is derived from glial cells, and is composed of lipid sheaths of cellular membranes wrapped around the axon of a neuron. Myelin improves the conduction of action potentials (APs), large (~100 mV), brief (<2 ms) depolarizations of the neural membrane, which are used to convey information to subsequent downstream neurons. AP conduction is improved by increasing the insulation of the axonal membrane, thereby elevating membrane resistance and decreasing capacitance. This allows the AP to travel along the axon with less loss of current through the membrane. APs propagate passively through myelinated portions of the axons, and are regenerated by active, voltage dependent Na^+^ and K^+^ ion-specific conductances at the intermittent, non-myelinated, nodes of Ranvier. This process is called saltatory conduction (Johnston and Wu, 1994; Figure 1).

Improved AP propagation by myelin has two important consequences for the animal: First, AP propagation becomes *energetically cheaper*, since APs only have to be regenerated at the nodes of Ranvier, not along the whole length of the axon. The cost of AP conduction in unmyelinated axons has been shown to be significant (Crotty et al., 2006). Second, myelin improves the *speed* of AP conduction (Rushton, 1951; Waxman, 1997). The trade-off between optimizing myelin for either of these advantages is at the center of the hypothesis presented here.

Myelin first evolved in the Ordovician, an epoch starting 488 ma ago (Bullock et al., 1984). While such minute soft tissues like axons never fossilize, we know this from comparing the nervous systems of extant animals. Jawless vertebrates (lampreys and hagfish), which split from the rest of the vertebrate lineages before the Ordovician, lack myelin (Bullock et al., 1984). As an evolutionary innovation, myelination co-occurred with jaws and the main sections of the vertebrate brain (forebrain, midbrain, hindbrain).

In the millions of years following the first appearance of myelin, vertebrates diversified enormously. However, this diversification was curtailed at the end-Permian mass extinction. This was the most severe mass-extinction so far recorded in the history of life on earth. An estimated >95% of marine species, and probably a comparable number of terrestrial species became extinct. The causes of this massive extinction are not completely clear with, ex., vulcanism, oxidation of carbon deposits, global warming, and ocean anoxia among the non-exclusive candidate mechanisms (Hallam and Wignall, 1997). After recovery from this mass-extinction event, the composition of the marine fauna significantly changed. Animals became more mobile, and the predation pressure among the fauna increased. This is inferred from the fossil record, which shows a steep decrease in the number of sessile species and an increase of mobile and actively predating species. In addition, multiple vertebrate and invertebrate lineages increased their predatory adaptions (crushing jaws) and anti-predatory defenses (armament, spines). Bore-holes in invertebrate shells became more common among other indications. This transition is referred to as the “Mesozoic Marine Revolution” (Vermeij, 1977). Long-term, large-scale escalatory trends in evolution have been suggested previously. A change in the role of myelin toward performance-enhancement (increased conduction speed) is in accord with such trends (Vermeij, 1987). The mesozoic marine revolution was probably not an abrupt event (Walker and Brett, 2002), but a qualitative change in marine ecosystem composition undoubtedly occurred. The more mobile, actively moving animals will have had different requirements for their nervous systems. The hypothesis we present here links the observed faunal changes with a proposed repurposing of the primary role of myelin.

Evolution is an optimization process constrained by the properties of biological tissues. A number of properties of the nervous system have also been explained by reference to optimization, such as the wiring of the cortex (Chklovskii and Koulakov, 2004), the scaling of cell numbers in the primate visual system (Stevens, 2001) and the shapes of dendritic trees in the insect visual system (Torben-Nielsen and Stiefel, 2010). These studies first determined where the theoretical optimum for a certain assumed goal lay. Then, they showed that the parameters in the real biological systems agree with the theoretical predictions, and hence these systems are likely to be optimized for the initially assumed goal.

While the optimization process in biological evolution is enormously complex and simulating it is out of question, we can numerically optimize models of myelinated axons. The trajectory toward the optima will be different from biological evolution, but the parameters of optimized myelinated axons will be quite conceivably very similar to those of real axons evolved toward the same set-point.

### The hypothesis

**Figure 2:**
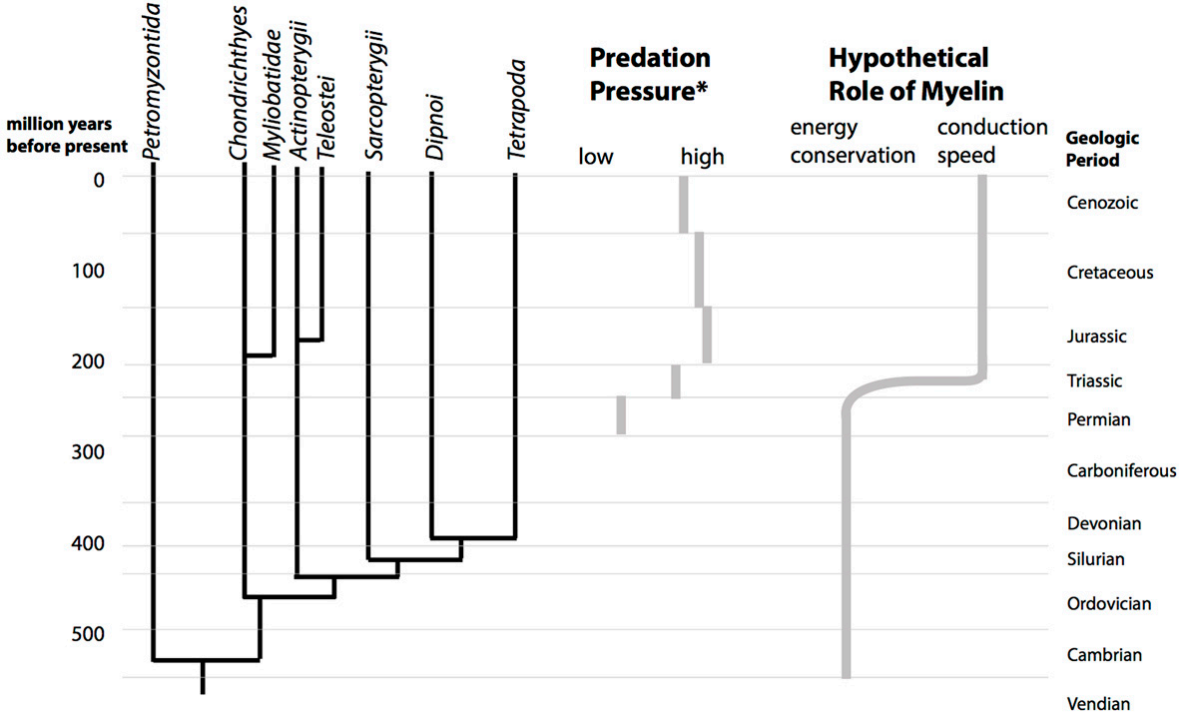
**The Mesozoic marine revolution and the proposed change in the role of myelin.** The cladogram shows the divergence of the vertebrate lineages over geological time (left). During the Mesozoic, starting in the triassic, the predation pressure in marine ecosystems increased (center). (^*^Predation pressure is estimated from durophagous (shell-crushing) predators, based on Figure 1 of Walker and Brett, 2002). Abundance of a vertebrate or invertebrate durophagous predatory lineage in a geological period was scored with 3 points, presence with 2 points and possible presence with 1 point. According to this estimate, predation pressure increases from a score of 9 in the Permian to a maximum of 20 in the Jurassic. We hypothesize that this upward shift in predatory pressure lead to a change in the structure myelin from being optimal for energy efficiency to being optimal for conduction speed (right).

Our hypothesis is that during the Mesozoic marine revolution, the role of myelin changed from mainly improving the energy efficiency of AP conduction to improving conduction speed. The more actively moving animals, which came to dominate ecosystems, used myelin for increasing the propagation speed of neural signals (Figure 2). This should equally true for predators as well as for the prey in need of improved escape behavior.

This hypothesis involves evolutionary changes of non-fossilizing tissues which happened 250 ma ago. Nevertheless, it has testable consequences. Specifically, it predicts that the axonal parameters will be located at different optima for different vertebrate lineages which were already separated *before* the Mesozoic marine revolution. In contrast, if the Mesozoic marine revolution and the subsequent increase in marine predation pressure did not cause a change in the role of myelin, then the parameters of myelinated axons should be relatively uniform in all vertebrate lineages.

Importantly, evolutionary optimization often does not follow a single objective, but a compromise. In the case investigated here, a trade-off between the usefulness of AP transmission speed and energy efficiency has to be found. To reformulate our hypothesis in this framework, we believe that the set-point of the evolutionary optimization process shifted on the continuum toward transmission speed during the Mesozoic marine revolution, and it shifted to different new set-points in different vertebrate lineages.

In the remainder of the paper, we first describe numerical optimizations of models of myelinated axons, optimized for a range of compromises between transmission speed and energy efficiency. We then look at the empirical literature for support of the hypothesis that the myelinated axons of different vertebrate lineages have parameters corresponding to different optima. Finally, we discuss future experimental work to further test our hypothesis, as well as the implications of our hypothesis for medical research.

## Methods

We first computed the optimal parameters of myelinated axons for low energy consumption and high conduction speed. Then, we compared the optimization results to the known parameters of vertebrate axons, and conclude with a discussion of myelin evolution.

### Optimization procedures

We have recently implemented the optimization approach outlined above based on applying genetic algorithms to models of neurons (Stiefel and Sejnowski, 2007; Torben-Nielsen and Stiefel, 2009). The details of the implementation of the optimization algorithm can be found in (Torben-Nielsen and Stiefel, 2009).

We performed optimization on the axonal parameters related to myelin to achieve the highest conduction velocity and the most energy-efficient conduction. By optimizing for both objectives simultaneously, we could analyze the trade-off between the axonal parameters and their influence on conductance velocity and energy efficiency.

More specifically, we constructed a simulation of an axon model in which the axonal parameters (Table 1) can be specified. We then ran a simulation in which we generate an AP in the attached soma and measure the conduction velocity. The efficiency is computed from the total amount of conductance, which is a good proxy for the total current flow, and hence, a good indication of energy expenditure.

**Table 1:** **Parameters of the axon model used in the multi-objective optimization for conduction speed and energy usage**.

**Parameter**	**Description**	**Limit**
No. nodes	Number of nodes of Ranvier	10–50
Node length	Length of the nodal segment	2–25 ⌈m
Node diam	Diameter of the nodal segment	5–15 ⌈m
Inter node length	Length of the inter-nodal segments	100,5000 ⌈m
Inter node diam	Diameter of the inter-nodal segments	2,25 ⌈m
Ra_node	Axial resistance of the nodal segments	80,120 ∧cm
Cm_node	Membrane capacitance of the nodal segments	0.6,1.4 F
g_leak_node	Leak conductance in the nodal segment	0.0003,0.03 Scm^-2^
g_Na_node	Peak sodium conductance in the nodal segments	0.12,2 Scm^-2^
g_k_node	Peak potassium conductance in the nodal segments	0.036,3.6 Scm^-2^
Ra_inter	Axial resistance of the inter-nodal segments	80,120 Ùcm
Cm_inter	Capacitance, myelinated inter-nodal membrane	0.0005,0.05 F
g_leak_inter	Leak conductance in the inter-nodal segment	1.5 10^6^,1.5 10^-4^ Scm^-2^

The limits are the values which the optimization algorithm was prevented from going above/below.

Optimization was done with a multi-objective genetic algorithm (Deb et al., 2002), an optimization technique loosely inspired by survival-of-the-fittest but in no way a copy of biological evolution. For such an optimization algorithm, an initial set of random axonal parameters was generated. Then, models of myelinated axons were generated from the parameter sets, and its performance was assessed according to the optimization objectives (velocity and energy-efficiency). We computed a fitness value jointly based on both objectives. Parameter sets and the models they represent which are better performing, with higher fitness values, were preferentially selected to remain in the pool of candidate models. These parameter sets were altered by mutation (random change of initial parameters) and recombination (reshuffling of parameters).

By iterating this process of assessing, selecting sets which are better than average, and mutating/recombining, the algorithm quickly yielded solutions which were performing the desired optimization criteria. The multi-objective variant of a genetic algorithm used here took two objectives (velocity and energy-efficiency) into account simultaneously. The best performing models displayed one of many optimal combinations of high conduction velocity and high energy-efficiency. The frontier of such models with optimal performance combinations is the Pareto-front.

Energetic cost was defined as the total charge and thus related to the total metabolic cost in one node: more charge offsets the ionic gradient more and thus the pumps needs to work more to achieve balance again (Sengupta et al., 2010). Velocity was computed from the time it takes for an AP to travel from one node to the next divided by the distance between the two nodes. We compute the AP velocity near the end of the axon where the AP shape has stabilized.

### Empirical data

We compared theoretically determined optima to empirical data, and a variety of measurements from a wealth of axons of vertebrates. (Additional data points will later be included as it becomes available in order to continue testing the theory).

### Types of data

The data acquired for each species should ideally contain information at least about conduction speed, axonal thickness, myelin thickness, inter-node distance, node length and sodium and potassium concentration at the node. The data should also be available for comparable axonal tracts, across species, from the central and PNSs. Measurements should include classic electron microscopy (for measuring lengths and diameters) and immuno-gold electron microscopy for measuring ion channel densities (Poliak and Peles, 2003). Electrophysiological recordings should provide information about AP conduction speed (Gillespie and Stein, 1983).

### Which species?

Ideally the compared species should be from different marine branches of the vertebrate cladogram (Figure 2). The main groups are the Chondrichthyes (cartilagenous fishes), the Actinopterygii (bony fishes), the Sarcopterygii (coelochants), Dipnoi (lungfishes) and Tetrapoda (land vertebrates). The Mesozoic change of predation pressure on land is less clear than in the ocean, but land vertebrates can at least serve as a control group. As stated above, we hypothesize that these groups will have different axonal/myelin parameter sets. Groups which are descending from a group of common ancestors alive during the Mesozoic share a set of axonal/myelin parameters. Thus, the cladogram constructed from the axon/myelin parameters should allow us to estimate the time of divergence of axonal functioning in vertebrates. Our prediction is that this divergence happened during the early Mesozoic.

## Results

### Numerical optimizations

**Figure 3:**
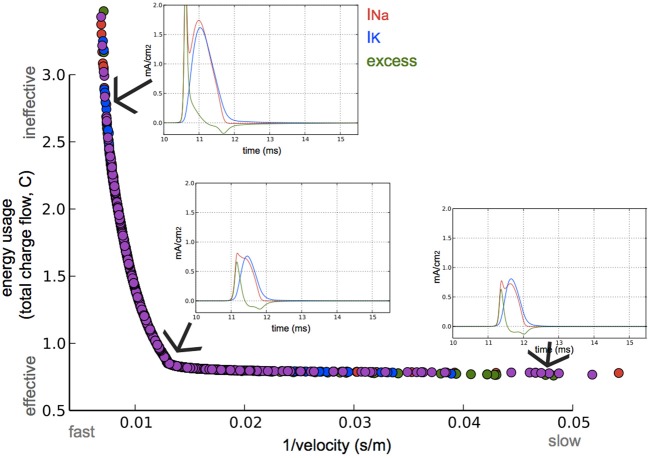
**Optimization for both high axonal conduction velocity and low energy consumption.** Speed and energetic cost for optimized models from four optimization runs (colors). Insets: Na^+^ and K^+^ currents, and the excess of Na^+^ current of the AP in a node at approximately three quarters the length of the axon. The 3 simulated models are optimized for different speed/efficiency trade-offs (taken from the points indicated by arrows).

The optimization algorithm we used yielded axonal models with parameter sets (within the bounds of their limits, Table 1) having the fastest APs with the highest efficiency possible. The performance of the resulting models in relation to these two objectives is shown in Figure 3. A clear trade-off can be observed between velocity and energy efficiency: some parameter sets give rise to very fast AP propagation (130 m/s) at a relative high cost (2.6, nearly 3x the cost of efficient ones), while energetically efficient parameter sets (0.8) give rise to slower AP propagation (55 m/s). The asymptotic convergence to a minimal energy is defined by the lower limit on the amount of g_na (0.12 mS/cm^2^) and g_k (0.036 mS/cm^2^).

**Figure 4:**
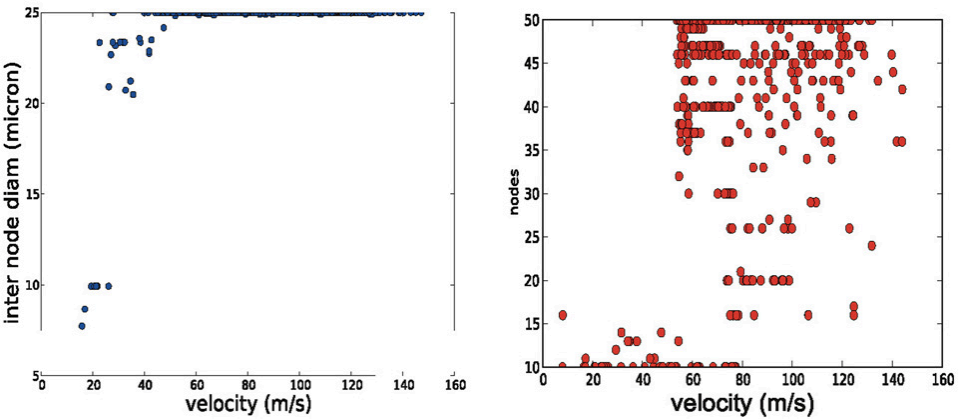
**Trends in axonal parameters related to conduction velocity.** Left conduction velocity vs. number of nodes. Right: conduction velocity vs. inter node diameter. Higher conduction velocities are achieved by more nodes of Ranvier and by larger axonal diameters.

The AP shape changed slightly and the amplitude of the energetically cheaper ones is lower (almost 20 mV). The axons optimized for fast AP propagation had the highest number of nodes, while the slower had fewer nodes but the exact number varies (10–40). A positive correlation exists between the number of nodes and the AP propagation velocity (Figure 4).

Both the Chondrichthyes and the Actinopterygii had already diverged into a multitude of lineages before the Mesozoic marine revolution. We hence don't know how many independent lineages separately transitioned the Mesozoic marine revolution. Thus, lineages which have only arisen during the Mesozoic will be of particular interest. Potentially, a single ancestral species later giving rise to these lineages transitioned the Mesozoic marine revolution. An example would be the rays (batoidea) among the Chondrichthyes and the modern bony fishes (teleostei), among the Actinopterygii, two groups which emerged during the Mesozoic (Long, 2010).

Due to several factors involving the difficulty of obtaining specimen and of modifying existing methods for animals of a lesser-used species, modern neurobiology is mostly concentrating on relatively few species (“model organisms”). Thus, the desired data are available from fewer species than is desirable, with a glaring lack of data from aquatic vertebrates. There is some published information along these lines for common model organisms such as rats and cats. We compiled a table (Table 2) with all the empirical data on the aforementioned axonal parameters found in the literature.

**Table 2:** **Parameters of myelinated axons in different vertebrate species and neural structures**.

Species:			Rat	Rat	Rat	Rana	Rana	Rabbit	Rat	Rana
Age:			adult	adult	adult	adult	adult	adult	adult	adult
Tissue type:			velum	velum	velum	sciatic		sciatic		
more specific:			IV tract	caudal	rostral					
Citation #:			1	1	1	2	3	4	5	6
myelin thickness		μ	4.35	3.8	2.94					
(μ m)		σ	1.88	1.85	1.14					
axon diameter		μ								
(μ m)		σ								
myelin + axon diam		μ				12–17				
(μ m)		σ								
gNa	S/cm2	μ								
		σ								
	number/μ m	channels				400–920		12000	2100	3000
	pS	single ch.				6.4				
gK	S/cm2	μ								
		σ								
	number	channels					570–960			
	pS	single ch.					2.7–4.6			
node length		μ								
(μ m)		σ								
internodal distance		μ	308	262	199					
(μ m)		σ	119	86	72					
conduction velocity		μ								
(m/s)		σ								
Species:			Mouse	Cat	Cat	Cat	Cat	Rat	Rat	Rat
Age:			adult	adult	adult	adult	adult	12 days	12 days	6 days
Tissue type:			sciatic	spine	sciatic	joint affe	pyramical	thora/spin	thora/spine	thora/spin
more specific:					a-motor			perip-seg	cent-seg	perip-seg
Citation #:			7	8	9	10	10	11	11	12
myelin thickness		μ	1.09							
(μ m)		σ								
										
axon diameter		μ	1.94		6.5					
(μ m)		σ	0.52							
										
myelin + axon diam		μ	3.03	2.8		1.6	0.8	40.3	22.5	25
(μ m)		σ	0.91					3.1	3.2	3.2
										
gNa	S/cm2	μ								
		σ								
	number/μ m	channels								
	pS	single ch.								
gK	S/cm2	μ								
		σ								
	number	channels								
	pS	single ch.								
node length		μ								
(μ m)		σ								
internodal distance		μ		300		107	87	167	78	167
(μ m)		σ						23	19	28
conduction velocity		μ			85					
(m/s)		σ								
Species:			Rat	Cat	Cat	Human	Human	Squirrel	Squirrel	Cat
Age:			6 days	21 days	10 yrs	newborn	adult	adult	Adult	adult
Tissue type:			thora/spine	CNS	CNS	CNS	CNS	CNS	CNS	CSN
more specific:			cent-seg	inf. Alv	inf. Alv	vent root	vent root	optic ner	Retina	sacral
Citation #:			12	13	13	14	14	15	15	16
myelin thickness		μ								
(μ m)		σ								
axon diameter		μ						1.2	0.6	1.3
(μ m)		σ								
myelin + axon diam		μ	11.7	4.2	9					
(μ m)		σ	1.5							
gNa	S/cm2	μ								
		σ								
	number/μ m	channels								
	pS	single ch.								
gK	S/cm2	μ								
		σ								
	Number	channels								
	pS	single ch.								
node length		μ								
(μ m)		σ								
internodal distance		μ	62	390	980	187	1500			93
(μ m)		σ	14			32				
conduction velocity		μ								6.7
(m/s)		σ								1.4
Species:			Dolphin	Whale	Mouse	Mouse	Cow	Pig	Dog	Cat
Age:			S. coeruleoalba	B. physalus						
Tissue type:			CNS	CNS	PNS	PNS	PNS	PNS	PNS	PNS
	more specific:		optic N	optic N	femoral	saphenous	phrenic	phrenic	phrenic	phrenic
Citation #:			17	17	18	18	19	19	19	19
myelin thickness		μ								
(μ m)		σ								
axon diameter		μ	2.45	1.98			7.4	7.4	6.5	7
(μ m)		σ					0.5	0.25	0.3	0.3
myelin + axon diam		μ								
(μ m)		σ								
gNa	S/cm2	μ								
		σ								
	number/μ m	channels								
	pS	single ch.								
gK	S/cm2	μ								
		σ								
	Number	channels								
	pS	single ch.								
node length		μ								
(μ m)		σ								
internodal distance		μ			1000	500				
(μ m)		σ								
conduction velocity		μ								
(m/s)		σ								
Species:			Rabbit	Rat	Mouse					
Age:										
Tissue type:			PNS	PNS	PNS					
more specific:			phrenic	phrenic	phrenic					
Citation #:			19	19	19					
myelin thickness		μ								
(μ m)		σ								
axon diameter		μ	5.3	4.6	4.6					
(μ m)		σ	0.2	0.17	0.6					
myelin + axon diam		μ								
(μ m)		σ								
gNa	S/cm2	μ								
		σ								
	number/μ m	channels								
	pS	single ch.								
gK	S/cm2	μ								
		σ								
	Number	channels								
	pS	single ch.								
node length		μ								
(μ m)		σ								
internodal distance		μ								
(μ m)		σ								
conduction velocity		μ								
(m/s)		σ								

Citations: 1, Ibrahim et al., 1995; 2, Sigworth, 1980; 3, Bengenisich and Stevens, 1975; 4, Ritchie and Rogart, 1977; 5, Chiu, 1980; 6, Dubois and Schneider, 1982; 7, Boyle et al., 2001; 8, Czarkowska et al., 1976; 9, Cullheim, 1978; 10, Deschenes and Landry, 1980; 11, Fraher, 1978a; 12, Fraher, 1978b; 13, Fried et al., 1982; 14, Friede at al., 1981; 15, Johnson et al., 1998; 16, Morgan, 2001; 17, Mazzatenta et al., 2001; 18, Ulzheimer et al., 2004; 19, Friede et al., 1984.

### Comparison between numerical optimizations and empirical data

We performed a literature study that is summarized in Table 2. Despite the spareness of the table, one can observe that both a higher number of nodes of Ranvier and larger inter-nodal diameter seem to be associated with higher conduction velocity in the axon. A similar connection can be found in our optimization results: higher propagation velocities are all obtained in models with the maximally allowed inter-node diameter (25 microns, Figure 4, left), and, with a higher number of nodes of Ranvier (Figure 4, right). Even if that the range of allowed inter-nodal diameters is larger (2–25 micron) in our optimization than reasonably found in nature, there is, nevertheless, a clear trend.

The available data is too sparse at this point to convincingly test the hypothesis that the role of myelin changed from energy conservation to propagation speed maximization. The empirical data lacks both complete information (all columns filled in Table 2) from one axonal tract, as well as phylogenetic diversity.

## Discussion

We hypothesize that during the Mesozoic marine revolution, the role of myelin changed from mainly improving the energy efficiency of AP conduction to improving conduction speed. This hypothesis has testable consequences, namely it predicts a phylogenetically biased distribution of axonal/myelin parameters. We conducted numerical optimizations of models of myelinated axons, and attempted to compare them to published information on myelinated axons. Unfortunately, the literature is too sparse, especially when it comes to non-model-organisms, and we cannot reach a definite conclusion about the state of our hypothesis. As of now, it is at least consistent, but not yet strongly supported by the empirical literature on myelinated axons.

Potentially, the peripheral nervous system (PNS) and the central nervous system (CNS) could operate along different speed/efficiency set-points. A number of time-dependent processes, such as the distinct types of oscillations seen in the CNS, could exert unique evolutionary pressures on conduction speed. On the other hand, the longer distances transversed by some PNS axons could exert pressure toward evolving higher conduction speeds as well. Better knowledge of the ontological changes in myelin and axonal conductance changes among the species included in the study would also help refine our understanding. In most cases, this kind of data is non-existent and the preponderance of the data included herein is from adult tissues.

An additional, extremely useful, piece of empirical information would be knowledge of the molecular biology of the ontogeny of myelin across multiple species. A comparison of the proteins involved in ensheathing axons with myelin, such as the oligodendrocyte myelin glycoprotein (Vourc'h and Andres, 2004), across lineages could reveal a time when these systems separated in vertebrates. Again, our hypothesis predicts a separation during the Mesozoic.

Furthermore, an evolutionary divergence of myelin during the Mesozoic marine revolution would have significant consequences for the use of different animal species as models for demyelination diseases. If the model species has myelin located at an optimum different from the human myelin-parameter optimum, its use as model species would be limited.

We hope that this hypothesis-paper serves as a motivation for our fellow neuroscientists to conduct measurements in less frequently investigated vertebrate species. The outcome of the testing of this hypothesis will have significant consequences for our understanding of vertebrate brain evolution. It will tell us if the Mesozoic marine revolution had macro-evolutionary consequences for vertebrate brains.

### Conflict of interest statement

The authors declare that the research was conducted in the absence of any commercial or financial relationships that could be construed as a potential conflict of interest.
